# An “All Teach, All Learn” Approach to Research Capacity Strengthening in Indigenous Primary Health Care Continuous Quality Improvement

**DOI:** 10.3389/fpubh.2018.00107

**Published:** 2018-04-30

**Authors:** Karen McPhail-Bell, Veronica Matthews, Roxanne Bainbridge, Michelle Louise Redman-MacLaren, Deborah Askew, Shanthi Ramanathan, Jodie Bailie, Ross Bailie, On Behalf of the Centre RCS Lead Group, Veronica Matthews

**Affiliations:** ^1^Poche Centre for Indigenous Health, Sydney Medical School, The University of Sydney, Camperdown, NSW, Australia; ^2^University Centre for Rural Health, The University of Sydney, Lismore, NSW, Australia; ^3^Centre for Indigenous Health Equity Research, CQUniversity, Cairns, QLD, Australia; ^4^College of Medicine and Dentistry, James Cook University, Cairns, QLD, Australia; ^5^Primary Care Clinical Unit, The University of Queensland, Herston, QLD, Australia; ^6^Southern Queensland Centre of Excellence in Aboriginal and Torres Strait Islander Primary Health Care (Inala Indigenous Health Service), Queensland Health, Inala, QLD, Australia; ^7^Hunter Medical Research Institute, University of Newcastle, Newcastle, NSW, Australia

**Keywords:** research capacity strengthening, continuous quality improvement, Indigenous leadership, research translation, primary health-care services, Aboriginal and Torres Strait Islander health, Indigenous health, collaborative leadership

## Abstract

In Australia, Indigenous people experience poor access to health care and the highest rates of morbidity and mortality of any population group. Despite modest improvements in recent years, concerns remains that Indigenous people have been over-researched without corresponding health improvements. Embedding Indigenous leadership, participation, and priorities in health research is an essential strategy for meaningful change for Indigenous people. To centralize Indigenous perspectives in research processes, a transformative shift away from traditional approaches that have benefited researchers and non-Indigenous agendas is required. This shift must involve concomitant strengthening of the research capacity of Indigenous and non-Indigenous researchers and research translators—all must teach and all must learn. However, there is limited evidence about how to strengthen systems and stakeholder capacity to participate in and lead continuous quality improvement (CQI) research in Indigenous primary health care, to the benefit of Indigenous people. This paper describes the collaborative development of, and principles underpinning, a research capacity strengthening (RCS) model in a national Indigenous primary health care CQI research network. The development process identified the need to address power imbalances, cultural contexts, relationships, systems requirements and existing knowledge, skills, and experience of all parties. Taking a strengths-based perspective, we harnessed existing knowledge, skills and experiences; hence our emphasis on capacity “strengthening”. New insights are provided into the complex processes of RCS within the context of CQI in Indigenous primary health care.

## Introduction

Globally, Indigenous peoples experience significant health disparities (1). Aboriginal and Torres Strait Islander (hereafter respectfully referred to as “Indigenous”) Australians experience disparities in health status and poor access to/quality of care when compared to non-Indigenous Australians (2, 3). Improving the quality of primary health care provided to Indigenous Australians is one necessary step to redress these health disparities (4). Continuous quality improvement (CQI) methods are key to improving primary health-care service delivery, with an emerging evidence base demonstrating promising results in Indigenous health (5, 6).

Systems and stakeholder capacity to participate in and lead CQI research in Indigenous primary health care is required to generate benefits (7, 8). Yet the processes, skills, knowledge, and system supports necessary to undertake CQI research in this setting have been largely undefined and fragmented. This raises the question of what is an appropriate model of CQI research capacity strengthening (RCS) in Indigenous primary health care that is of value and benefit to Indigenous Australians?

The *Centre for Research Excellence in Integrated Quality Improvement for Indigenous primary healthcare services* (the Centre) has collaboratively developed a CQI-RCS model. This model draws on capacity strengthening (9) and quality improvement capacity building evidence (10), to theorize strategies to strengthen capacity for CQI research in Indigenous primary health care. Reflecting extant capacities, loci of power, cultural strengths and relationships, the model aims to strengthen existing capacity rather than assume a need to “build” from a zero base (11); as reflected in the Centre’s “all teach, all learn” approach to CQI-RCS. This paper presents the model’s development and four principles to inform approaches to Indigenous health RCS.

## CQI Research in Indigenous Health

Continuous quality improvement refers to “a system of regular reflection and refinement to improve processes and outcomes that will provide quality health care” (12). Through CQI research, primary health-care quality improvement efforts have been accelerated and strengthened at multiple levels and contexts (13). Clinical performance assessment and improvements have been achieved across a range of services (14, 15) and structured health service and systems assessment facilitated to support best practice (16, 17). CQI research continues to play an important role in testing the acceptability of CQI approaches and their impact on Indigenous primary health care (5, 18).

In Australia, CQI methods have attracted significant resourcing and policy focus, including for Indigenous primary health care. The *National CQI Framework for Aboriginal and Torres Strait Islander Primary Health Care* and the National Aboriginal Community Controlled Health Organisation both prioritise CQI-RCS in the Indigenous community-controlled sector to support co-creation and translation of CQI knowledge (19, 20). The National Health and Medical Research Council funded the Centre for 2015–2019 to improve Indigenous health outcomes by accelerating and strengthening system-wide CQI efforts. The Centre brings together leaders and practitioners of CQI in Indigenous primary health care from community-controlled and government health services, universities, research centers, and policy organizations across Australia (21).

## Health RCS

The capacity to perform research is an essential component of a well-functioning public health system (22). A variety of research capacities are required to lead and/or participate in health research aimed at individual, organizational, and systems-level change (23). RCS occurs through research processes and systems, including but going beyond individual training when health equity is the goal (11). Health RCS involves skills- and confidence-building through training, mentorship triads, and apprenticeship-style learning; partnerships and collaborations; dissemination and knowledge translation; empowerment, local governances (ownership) and leadership; as well as infrastructure and resources (24). A literature review by Kahwa and colleagues defined health RCS as:

An ongoing and iterative process of empowering individuals, interdisciplinary teams, networks, institutions and societies to identify health and health-related challenges; to develop, conduct and manage scientifically appropriate and rigorous research to address those challenges in a dynamic and sustainable manner; and to share, apply and mobilize research knowledge generated with the active participation of engaged stakeholders and decision-makers (25).

This definition underscores the need for a systems-orientation and the importance of empowerment and capacity in research production and utilization. The Kahwa definition does not detail specific capacities to be strengthened through RCS. The definition does, however, identify a framework in which RCS occurs within and across five structural levels: individual, team, health services, network and support, and national research infrastructure/bodies (25). Eight integrated dimensions for health RCS design, coordination and evaluation span the five levels (25):

1. building skills and confidence
2. research applicability
3. developing partnerships and linkages
4. appropriate dissemination and knowledge translation to maximize impact
5. including elements of continuity and sustainability
6. making investments in infrastructure to enhance research capacity building
7. leadership
8. empowerment

## Indigenous Health RCS

The backdrop to Indigenous health RCS is a history of imposed research that generally has not benefited Indigenous Australians, reflecting the exploitative nature of colonization in Australia (7, 26). We acknowledge there are positive examples of Indigenous health research. Yet historically research was about examining and documenting the “exotic other” and conducted to protect non-Indigenous populations from disease (27). More recently, it has often served the priorities of non-Indigenous researchers and perpetuated a deficit-narrative that justifies mainstream intervention in Indigenous people’s lives “for their own good” (28). Such an approach undermines Indigenous control—an important determinant of population health and wellbeing (29)—while overlooking the value and strengths of Indigenous knowledge, experience, and perspectives (30).

Indigenous health RCS is important for self-determination of health research and sustainable outcomes for Indigenous people (31, 32). Research led by Indigenous people and communities provides a means of re-asserting control over country, livelihoods, and knowledge impacted through colonization (33). Indigenous research leadership has been changing the narrative so that Indigenous paradigms, community needs, priorities, and culture are at the center of health research, and designed to deliver benefit to Indigenous peoples (8). Capacity development of all involved in Indigenous health research remains a crucial factor for successfully developing this new research paradigm (8).

One 5-year Indigenous RCS intervention found that keys to success were: an agreement to embrace Indigenous research principles with creation of space for two-way learning between Indigenous and non-Indigenous researchers; recognizing Indigenous ownership, leadership and respect as core to an Indigenous research agenda; and acknowledging “cultural difference challenges” inherent to relationships between Indigenous and non-Indigenous researchers (34). As such, Indigenous health RCS must involve mutual learning (35)—“all teach, all learn”—and a strengths-based approach to Indigenous health research that embraces concepts of Indigenous wellbeing, knowledge and sovereignty (30, 36, 37).

## “All Teach, All Learn” for CQI-RCS in Indigenous Primary Health Care

The Centre developed and implemented a dedicated CQI-RCS Program, which intersects with its network and research programs, and seeks to enhance capacities within the Indigenous primary health-care setting to conduct and use CQI research. The Centre’s motto, “all teach, all learn” embodies the value placed upon mutual learning, where CQI-RCS Program users are both learners and teachers. CQI-RCS Program users include Indigenous and non-Indigenous:

- research translators: service providers, managers, policy makers, and communities in areas relevant to comprehensive primary health care in Indigenous communities.
- early-career researchers: post-graduate and undergraduate students; research project officers; Ph.D. candidates; early-career, and mid-career researchers.
- Senior researchers: more senior researchers with experience in research on CQI, comprehensive primary health care, health systems, and other related areas.

## Developing the CQI-RCS Model

The collaborative development of the CQI-RCS model commenced with the appointment of a full-time RCS fellow in 2016, followed by establishment of a RCS Lead Group (Lead Group), development of a values and ethics protocol (Table 1), and construction of a program logic to guide the embedding of the CQI-RCS Program across the Centre’s activities.

**Table 1:** Values and ethics protocol for continuous quality improvement (CQI) research capacity strengthening (RCS).

This protocol for operation was endorsed in January 2017
Reflecting feedback at the Centre 2016 network meeting in Darwin and the 2016 Lowitja Conference statement (38), the Centre CQI-RCS Program recognizes the need for reflexive dialogue to examine how ethical principles in Indigenous health research are applied in the Centre RCS program and how researchers and community stakeholders can navigate this terrain (39). To contribute to this dialogue, the Centre CQI-RCS Program draws on the Values and Ethics: Guidelines for Ethical Conduct in Aboriginal and Torres Strait Islander Health Research (40) to inform its ethical operation, alongside the Centre principles of practice (see end of table).

***Reciprocity***

Reciprocity requires the Centre CQI-RCS Program to demonstrate a return or benefit to users that is valued and which contributes to inclusion, cohesion and survival of Aboriginal and Torres Strait Islander people.
Inclusion:
draw from and prioritize Indigenous scholarship to shape the Centre RCS Program. seek to establish relationships and mechanisms to ensure Aboriginal and Torres Strait Islander leadership and direction of the Centre CQI-RCS Program, including the RCS Lead Group. establish and nurture relationships with Aboriginal and Torres Strait Islander Centre members and partners (including potential) for involvement in the Centre CQI-RCS Program.
Benefit:
through the 2017 CQI-RCS program logic workshop, clarify the potential benefit of the Centre CQI-RCS program. collaboratively develop a research grant application to developmentally assess CQI-RCS in the Centre, using existing indicators and those emerging through the CQI-RCS activities. prioritize Aboriginal and Torres Strait Islander researchers for Centre scholarships.

***Respect***

Respect acknowledges the individual and collective contribution, interests, and aspirations of Aboriginal and Torres Strait Islander people, researchers, and other partners in the research process.
Respect people and their contribution:
acknowledge Aboriginal and Torres Strait Islander people involved in the CQI-RCS Program as co-generators of knowledge regarding CQI-RCS, including authorship on materials produced.
Minimize difference blindness:
create mechanisms through the Centre CQI-RCS program that draw attention to decisions by and engagement of Aboriginal and Torres Strait Islander people in the Centre. utilize or build upon existing Aboriginal and Torres Strait Islander structures where they exist, including by engaging with peak representative bodies.
Recognize the consequences of research:
seek to establish agreement within the Centre regarding CQI-RCS program deliverables, including consideration to the pre-existing program and potential future proposals. Include discussion regarding who needs to be involved, how and when in the processes, from the beginning, including “getting the research question right.” respect that there are some boundaries beyond which non-Indigenous researchers cannot go and that Aboriginal and Torres Strait Islander researchers and partners may choose at any time, for any reason without consequence, to cease their involvement with the Centre CQI-RCS program.

***Equality***

To enact equality:
Value knowledge and wisdom:
seek multiple input points for Aboriginal and Torres Strait Islander researchers and partners into the Centre CQI-RCS program design processes, delivery and outcomes. following the Centre principle, Respect the past and present experiences of Aboriginal and Torres Strait Islander people, appreciate Aboriginal and Torres Strait Islander peoples’ collective memory and shared experience in data collection and analysis.
Seek equality of partners:
seek to establish mechanisms in the Centre CQI-RCS program that ensure Indigenous leadership and direction of research, such as the RCS Lead Group.
provide regular updates on the Centre CQI-RCS Program, which can include sharing information, documents, tools, and presentations generated through the Centre CQI-RCS Program. adjust research processes and directions as seen necessary for equality. For example, should there be an Aboriginal and Torres Strait Islander researcher/s interested to co-lead the Centre CQI-RCS Program, seek to enable this partnership. acknowledge that Aboriginal and Torres Strait Islander autonomy and participation are core to ethical research in Indigenous studies (41). Aboriginal and Torres Strait Islander people have been long calling for reform of research (7, 26, 33, 42), for which it is essential that research relating to Indigenous people is carried out on their terms, led and directed by Indigenous people (43).
Distribute the benefits:
seek to ensure that the Centre CQI-RCS program delivers benefits that are of equal value to Aboriginal and Torres Strait Islander researchers just as much as to Centre Chief Investigators and lead researchers. pending interest, negotiate co-authorship with Aboriginal and Torres Strait Islander people involved, where the opportunity arises to co-publish.

***Responsibility***

Responsibility involves doing no harm and establishing processes for accountability, for which the Centre CQI-RCS Program and its team will:
seek to attend Centre and other meetings (upon invitation) to provide opportunity for partners to discuss and query the Centre CQI-RCS program. regularly update on Centre CQI-RCS Program progress (e.g., monthly email update, RCS Lead group meetings), ensuring scope for Aboriginal and Torres Strait Islander to correct and influence activities and priorities. abide by the ethics approval for the Centre, when the Centre CQI-RCS Program involves research activity. where there is intention to publish in relation to the Centre CQI-RCS program, ensure that agreement has been reached by obtaining formal approval by the Centre for publication. draw on scholarship and transformative agendas advocated for by Aboriginal and Torres Strait Islander scholars, to inform the Centre CQI-RCS Program (33, 34, 44).

***Survival and protection***

Survival and protection acknowledges the importance of values based solidarity to Aboriginal and Torres Strait Islander peoples; respect for social cohesion; and commitment to cultural distinctiveness. The Centre CQI-RCS Program will endeavour to enact this by the following strategies
Seek to safeguard against discrimination or derision of Aboriginal and Torres Strait Islander identity and cultural diversity, including consultation with Aboriginal and Torres Strait Islander researchers involved in the Centre in identifying CQI-RCS needs, as well as to identify threats and ways to eliminate those threats. Work according to a strengths-based approach (45). Utilize the RCS Lead Group, advisors, mentors, and critical friends for guidance to work in a way that progresses the jointly identified CQI-RCS agenda.

***Spirit and integrity***

Protecting the spirit and integrity of Aboriginal and Torres Strait Islander communities and individuals is an obligation for Centre researchers, which in the Centre CQI-RCS Program involves:
motivation and action: through the above-mentioned mechanisms, seek to remain transparent and consistent with Aboriginal and Torres Strait Islander values, such as those outlined here. intent and process: seek to negotiate proposal designs for CQI-RCS activities and implementation of the CQI-RCS Program with Aboriginal and Torres Strait Islander researchers, including using workable timeframes and decision-making processes.
**The Centre Principles of Practice^a^:**
respect the past and present experiences of Indigenous people. work in partnership. ensure Indigenous leadership and direction of research. ethical conduct. get the research question right. design research that will be feasible, produce outcomes and build capacity. identify and provide the right resources and training. establish systems and practices to support the application of evidence to improve Indigenous primary health care and health outcomes.

*^a^We acknowledge that these principles of practice were generously shared with the Centre, by the Centre for Research Excellence in Discovering Indigenous Strategies to Improve Cancer Outcomes Via Engagement, Research Translation and Training (DISCOVER-TT). More information available here: https://www.menzies.edu.au/page/Research/Centres_for_Research_Excellence/Centre_for_Research_Excellence_Cancer/*.

### The Lead Group

The Lead Group, established in January 2017 by the non-Indigenous RCS Fellow (Karen McPhail-Bell) and co-chaired by Veronica Matthews, an Aboriginal researcher, guided the development of the CQI-RCS Program (see Terms of Reference in Supplementary Material). The Lead Group’s composition reflected systems-based participatory action research (21) and triads for enhancing RCS (hubs of research translators, early-career and senior researchers) (46). Members included Indigenous and non-Indigenous early-career and senior researchers, government and community-controlled health service representatives, who met monthly by teleconference during the development phase (see Acknowledgments). The Lead Group operated within the governance structure of the Centre, with co-lead representation at quarterly Centre Management Committee meetings.

### Values and Ethics Protocol

The Lead Group developed a values and ethics protocol to express how the CQI-RCS Program would address the six values of ethical research with Indigenous people and communities (47) (Table 1). The protocol embodied the “all teach, all learn” maxim, with Indigenous members mentoring non-Indigenous members (and *vice-versa*), and senior researchers mentoring less experienced researchers. The protocol provided direction for the Lead Group’s approach to shared decision-making and activities, including creating space for dialog regarding matters of power and control over the CQI-RCS program.

### CQI-RCS Program Logic Development Process

A program logic describes how a program’s activities, impacts, outputs, and outcomes interact, to show the intended causal links (48). Development of the CQI-RCS Program logic sought to enact a collaborative partnership approach that, given the Indigenous health research context (described above), focused on the lived experiences, ideas, interests, and aspirations of Indigenous people (49, 50). Development occurred in four phases. First, through examination of key literature, particularly Indigenous scholarship in this space, and reflecting a strengths-based approach (45, 51), the Lead Group defined CQI-RCS as follows.

CQI-RCS means to enhance capacities to conduct and use CQI research that is valued by and of benefit to Indigenous peoples, in the Indigenous primary healthcare setting, with the specific purpose of supporting integrated quality improvement and building on the collaborative platform of the Centre.

CQI-RCS acknowledges existing strengths, knowledge and work. Through “all teach, all learn”, CQI-RCS involves a mutual exchange between Indigenous and non-Indigenous researchers, research users, and communities to ensure sustained benefit from CQI research. CQI-RCS enables individuals, communities, organizations, services and broader systems to make informed decisions about, participate in, utilize, lead and generate CQI research.

Second, the Lead Group sought feedback from all members (approximately 70 individuals) of the Centre’s network regarding: current CQI-RCS successes and challenges; suggestions to support Indigenous leadership and participation in CQI research; and short-term and long-term outcomes that could inform the development of the program logic and the CQI-RCS Program.

Third, the Lead Group, Centre research-leads and invited Indigenous leaders in RCS came together for a 1-day workshop, designed and facilitated by Karen McPhail-Bell and Veronica Matthews with the Lead Group, to develop a CQI-RCS program logic model. Fourth, the Lead Group presented the model to the Centre’s bi-annual network meeting for feedback, prior to finalization.

**Figure 1:**
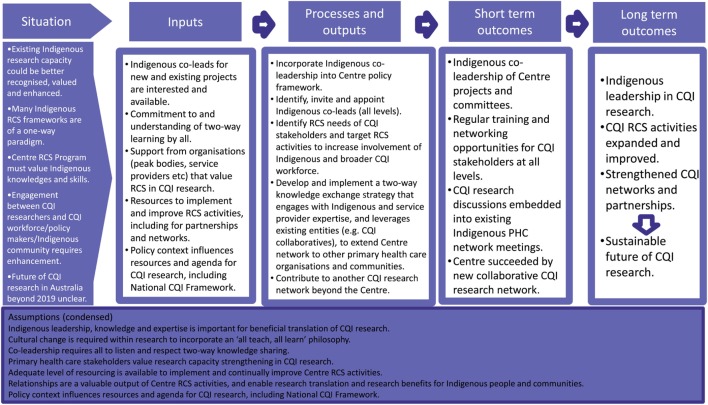
An overarching program logic “map” of the Centre’s Continuous Quality Improvement Research Capacity Strengthening Program.

The program logic identifies CQI-RCS program priorities for implementation tied to short- and long-term outcomes, activities, participation, input, assumptions, and monitoring and evaluation indicators. The priorities are: indigenous co-leadership in the Centre’s CQI research; improve and expand the Centre’s RCS activities; strengthen CQI research networks and partnerships; and develop sustainable CQI research for improving Indigenous health through primary health care. A detailed program logic was produced for each of these priorities, which tied together in one overarching program logic map (Figure 1). The program logic model encapsulates the value of Indigenous-led CQI research for strengthening primary health care, to benefit Indigenous peoples and improve Indigenous health.

## Guiding Principles for an “All Teach, All Learn” Approach to CQI-RCS

Four principles informed the CQI-RCS approach and provide a possible starting point for those seeking to implement RCS models in Indigenous primary healthcare contexts. The principles are discussed in detail below: (1) mutual/two-way learning; (2) Indigenous co-leadership as core; (3) sharing power and facilitating relationships; and (4) resourcing and continuous improvement.

### Principle 1: Mutual/Two-Way Learning

At the core of the “all teach, all learn” motto is the valuing of Indigenous cultures, knowledge, and expertise alongside Western research and knowledge, and recognition that different kinds of capacities are to be developed in different people, processes, organizations, and systems. For example, in the Indigenous primary health-care context, individual capacities include knowledge, skills, and experience to: conduct and critically assess research; participate in all stages of the research; contribute to research-informed action; maintain respectful relationships; and facilitate culturally safe processes. Consistent with other mutual RCS approaches, we found that not all the same capacities need to be developed in all stakeholders, and that such capacities are influenced by worldview, knowledge, experience, and relationships (11, 34). The Lead Group’s approach was informed by the collective understanding that in the Australian context, mutuality requires valuing Indigenous voices, identity and knowledge, and recognition of power held by decision-makers, institutions, and structures to improve or undermine Indigenous health (35, 52–56).

### Principle 2: Indigenous Co-Leadership as Core to Indigenous Health Research

Indigenous-led research refers to research that is led and driven by Indigenous researchers, practitioners, policy makers, and communities in partnership with community organizations or through collaborative approaches involving Indigenous community/ies at each stage of the research process (53). As a step toward Indigenous leadership, the CQI-RCS model seeks to enact Indigenous co-leadership with non-Indigenous people already holding leadership roles. Across the Centre’s research projects, non-Indigenous leads have/are working to establish co-leadership arrangements with Indigenous people involved in those projects, in recognition of the capabilities and expertise brought by Indigenous people to the research process. Co-leads can be researchers, community members, service providers, and policy makers of varying seniority or position, who support mutual RCS. These co-lead arrangements involve attention to creating culturally safe spaces, as reflected in the values and ethics protocol (Table 1).

### Principle 3: Sharing Power and Facilitating Relationships

A commitment to Indigenous co-leadership brings considerations and tensions to negotiate collectively, to produce mutually beneficial outcomes. Methodologies that seek to support and value Indigenous knowledge within CQI research, and involve Indigenous people and their interests, are necessary (57). For non-Indigenous people in leadership roles, this means leading in partnership with Indigenous people and organizations, and seeking to share power over ownership of the research and associated decisions, processes, outcomes, and benefits.

The Lead Group provided a structure to enable Indigenous co-leadership of CQI-RCS and share decision-making and direction between diverse individuals and perspectives. The development process collectively identified and formalized the CQI-RCS priorities and approach, which provided momentum for power-sharing and involvement of more Indigenous people within the Centre. Respectful relationships underpinned these processes and structures, as reflected in the program logic (Figure 1). Strengthening networking and partnerships for CQI-RCS are key for transfer and implementation of innovations (58), and accountability and research priorities that benefit Indigenous Australians (7). Indigenous ownership and stakeholder relationships from the outset of research enhance the likelihood of research relevance and thus translation and benefit (43). Allowing sufficient time for meaningful relationship building is essential for quality research in this space and is a RCS activity itself as it enables respectful engagement with Indigenous knowledge and perspectives (26, 34, 59).

### Principle 4: Resourcing and Continuous Improvement

Resources (especially staffing) are required to enable the communication, relationships, engagement and training that facilitate CQI-RCS. Likewise, resourcing and continuous learning are essential to enable co-leadership, which is generally an additional responsibility for busy Indigenous translators/leaders/researchers. While the Lead Group developed the CQI-RCS program model, implementation will be the responsibility of all within the Centre. Using a continuous improvement approach, the Centre can now test application of the CQI-RCS program logic across its research programs and activities and monitor progress against the CQI-RCS priorities.

## Connection to Other RCS Frameworks

CQI-RCS must occur across multiple domains and levels to enable broader, systems-level engagement in health research at all stages, from research question formation to dissemination of outcomes. Our multi-level, systems view of CQI-RCS is consistent with other RCS models, such as that of Kahwa et al. (25) (described earlier) with integrated dimensions at individual, team, health service, network, and national levels. Our model adds to this by including the requirement that CQI-RCS is contextualized according to Indigenous leadership, knowledge, and aspirations, with principles to guide an approach to CQI-RCS.

As stated earlier, our model draws on generic and quality improvement capacity building evidence. Like quality improvement capacity building (10), knowledge is limited regarding methods to attribute successful elements to RCS interventions (25). The numerous RCS definitions in the literature (23, 60–63) are reported to have adversely affected development of meaningful assessment, monitoring, and evaluation of RCS; so has the context-specific and complex nature of RCS (25). The CQI-RCS principles presented here provide a pathway to address this knowledge gap, through implementation and evaluation in other contexts.

## Limitations and Strengths

We acknowledge that while program logics have considerable merits, their linear and managerial format can be alienating, lack a systems perspective, and neglect relationships between people and process issues (64, 65). Some program logics include feedback loops to counter the typical linear approaches (66). Like other researchers (67, 68), we consider our program logic (Figure 1) to be an approximation of an anticipated path to be adjusted over time, rather than a replica of reality.

A strength of this CQI-RCS model is its emphasis upon inclusivity of voices and co-leadership, which is reflected in its collaborative development. A traditional, Western approach to research does not convey the model’s espoused principles of power-sharing, and Indigenous and non-Indigenous co-leadership. We are conscious that Indigenous health RCS may have produced unintended consequences of non-Indigenous researchers benefiting (through grants, publications, etc.) at the expense of Indigenous-led research (44, 69). However, these tensions are not reason to discontinue involvement of non-Indigenous researchers; nor do we believe this CQI-RCS work has been at the expense of Indigenous-led research. Rather, non-Indigenous researchers must uphold the struggle for genuine self-determination of Indigenous people and engage with Indigenous people as thinkers, knowers, and experts (30). Our model, with its guiding principles and logic, provides a tool to guide RCS in Indigenous primary health-care contexts, to enable sustained and Indigenous-controlled health research that benefits Indigenous people and communities, framed within a CQI and systems approach.

## Conclusion

This paper presents the development of a CQI-RCS model in Indigenous primary health care. The program logic provides a framework for CQI-RCS implementation, monitoring and evaluation of collaboratively determined priorities and outcomes. This is important because there are no existing models of which we are aware that articulate and inform CQI-RCS in Indigenous primary health care in this way. The model also points to the importance of mutual learning, Indigenous co-leadership, power-sharing and sufficient resourcing. The new knowledge generated through developing this model echoes calls of Indigenous Australians seeking research reform that benefits Indigenous peoples (7, 26, 33, 57). The CQI-RCS model presents a possible pathway to enhance capacities to conduct and use CQI research that is valued by and of benefit to Indigenous peoples in the Indigenous primary health-care setting.

## RCS Lead Group Members

Co-Lead: **Veronica Matthews**, Research Fellow, Wingara Mura Leadership Program Fellow, The University of Sydney, University Centre for Rural Health; Co-Lead: **Karen McPhail-Bell**, Research Capacity Building Fellow, University of Sydney, University Centre for Rural Health; **Kerry Copley**, CQI Program Coordinator—Top End, Aboriginal Medical Services Alliance NT (AMSANT); **Louise Patel**, CQI Program Coordinator—Central Australia and the Barkly, AMSANT; **Roxanne Bainbridge**, Principal Research Fellow—Indigenous Health, School of Health, Medical and Applied Sciences, CQUniversity; **Michelle Redman-MacLaren**, Adjunct Academic, Centre for Indigenous Health Equity Research, CQUniversity; Senior Research Fellow, College of Medicine and Dentistry, James Cook University; **Deborah Askew**, Research Director, Southern Queensland Centre of Excellence in Aboriginal and Torres Strait Islander Primary Health Care (Inala Indigenous Health Service); **Shanthi Ramanthan**, Post Doctorate Fellow—Health Research Economics, Hunter Medical Research Institute; **Nalita Turner**, Researcher, Menzies School of Health Research; **Ross Bailie**, Director, University of Sydney, University Centre for Rural Health—North Coast; CIA the Centre; **Jodie Bailie**, Research Fellow (Evaluation), University of Sydney, University Centre for Rural Health; **Isaac Hill** (January–April 2017), Statewide Data and IT Coordinator—CQI Unit, Aboriginal Health Council of SA; **Janya McCalman** (January–April 2017), Senior Research Fellow, School of Health, Medical and Applied Sciences, CQUniversity.

## Author Contributions

KM-B conceived, drafted, reviewed, and finalized the manuscript for submission. All authors critically revised and approved this manuscript and, with the Lead Group (named in Acknowledgements), provided co-leadership for the Centre CQI-RCS work that forms this manuscript’s content.

## Conflict of Interest Statement

The authors declare that the research was conducted in the absence of any commercial or financial relationships that could be construed as a potential conflict of interest.
