# Supplementary material for: An “All Teach, All Learn” Approach to Research Capacity Strengthening in Indigenous Primary Health Care Continuous Quality Improvement
Source: Front Public Health. 2018 Apr 30;6:107. doi: 10.3389/fpubh.2018.00107 (PMC5936797; doi:10.3389/fpubh.2018.00107)
Supplement: Supplementary file 1 [file presentation_1.PDF]

Supplementary Material

Terms of reference to establish the Centre RCS Lead Group January 2017

Karen McPhail-Bell with the Centre RCS Lead Group

*** Correspondence:** Corresponding Author: karen.mcphail-bell@sydney.edu.au

1 Introduction

This document supports the establishment of the *Centre for Research Excellence in Integrated Quality Improvement for Indigenous Primary Healthcare* (the Centre) Research Capacity Strengthening (RCS) Lead Group (hereafter referred to as the Lead Group) in January 2017, including identification of potential members, and is to be read in conjunction with the Centre RCS 2017 Plan (1).

2 Lead Group aim

In its draft RCS Program Plan (2), the Centre outlined its intention for the Lead Group as being to "take responsibility for refinement and implementation of the research capacity building plan". In the RCS 2017 Plan, this responsibility also includes a role in the development and monitoring of RCS indicators (1).

3 Proposed Lead Group tasks for 2017

The following tasks are proposed areas of focus for the Lead Group in 2017. Tasks and the RCS Program focus may evolve once the group is established and active in clarifying its priorities.

- Develop a protocol for operation that ensures Indigenous leadership and direction of the RCS Program, and reciprocal two-way benefit.
- Establish agreement within the Centre as to what the RCS Program will deliver, including development of a definition of RCS.
- Provide advice regarding the priorities and design of grant application/s by the RCS Fellow in relation to the RCS program.
- Provide guidance in the design and implementation of a collaborative RCS program logic/indicator workshop.
- Provide ongoing guidance regarding the assessment and refinement of RCS indicators during the life of the Centre, to guide planning, monitoring and evaluation of RCS program.

4 Proposed RCS Lead Group membership

Membership of the Lead Group could include available, pre-identified members as outlined in the draft Centre RCS Program Plan (2) and others with expertise in RCS who have since indicated interest to support refinement and implementation of the RCS Program.

RCB Lead Group Role	Name	Organization	Aboriginal and/or Torres Strait Islander?
Co-chair	Nomination To Be Confirmed		Yes
Co-chair	Karen McPhail-Bell	The University of Sydney	No
Researcher & Centre Evaluation Team perspective	Roxanne Bainbridge	CQUniversity	Yes
Researcher/Centre Associate Investigator (AI)	Janya McCalman	CQUniversity	No
Researcher	Nalita Turner	Menzies School of Health Research	Yes
Researcher	Veronica Matthews	Menzies School of Health Research	Yes
Aboriginal Community Controlled Health Organization (ACCHO) peak body perspective/Centre AI	Louise Patel/ Kerry Copley	Aboriginal Medical Services Alliance Northern Territory	No
Government Indigenous health service perspective/Centre AI	Deb Askew	Inala Indigenous Health Service	No
ACCHO peak body perspective	Isaac Hill	Aboriginal Health Council of SA	Yes

5 Frequency of meetings

It is anticipated that the Lead Group will meet bi-monthly with communication between meetings.

6 References

1. McPhail-Bell K. CRE-IQI Research Capacity Building Program: 2017 Plan. Sydney: Centre for Research Excellence in Integrated Quality Improvement in Indigenous Primary Health Care Services; 2016. p. 1–5.

2. Bailie R. CRE-IQI Research Capacity Building Program Plan DRAFT. Brisbane: Menzies School of Health Research; 2015.
